# Mechanistic studies of uncatalyzed and ruthenium(III)-catalyzed oxidation of the antibiotic drug chloramphenicol by hexacyanoferrate(III) in aqueous alkaline medium: a comparative kinetic study

**DOI:** 10.1007/s00706-014-1208-7

**Published:** 2014-05-24

**Authors:** M. D. Meti, K. S. Byadagi, S. T. Nandibewoor, S. A. Chimatadar

**Affiliations:** P. G. Department of Studies in Chemistry, Karnatak University, Pavate Nagar, Dharwad, 580003 India

**Keywords:** Chloramphenicol, Hexacyanoferrate(III), Ruthenium(III) catalysis, Oxidation, Thermodynamic parameters

## Abstract

**Abstract:**

The kinetics of oxidation of the antibiotic drug chloramphenicol (CHP) by hexacyanoferrate(III) (HCF) has been investigated spectrophotometrically both in the absence and presence of ruthenium(III) catalyst in aqueous alkaline medium at 25 °C and at constant ionic strength of 1.10 mol dm^−3^. The stoichiometry is identical in both cases, i.e. [CHP]/[HCF] = 1:2. The oxidation products were identified by TLC and spectral studies such as GC–MS, IR, and ^1^H NMR. In both catalyzed and uncatalyzed reactions, the order with respect to the concentration of HCF is unity, whereas the order with respect to the concentration of CHP and the concentration of OH^−^ is less than unity over the concentration range studied. The order with respect to the concentration of Ru(III) is unity. The reaction in the presence of Ru(III) is approximately tenfold faster than the uncatalyzed reaction. The active species of oxidant and catalyst are [Fe(CN)_6_]^3−^ and [Ru(H_2_O)_5_(OH)]^2+^, respectively. On the basis of experimental results suitable mechanisms are proposed. The reaction constants involved in the different steps of the reaction mechanisms were calculated for both cases. The catalytic constant was also calculated for the catalyzed reaction at different temperatures. The activation parameters with respect to the slow step of the mechanism and thermodynamic quantities are also determined.

**Graphical abstract:**

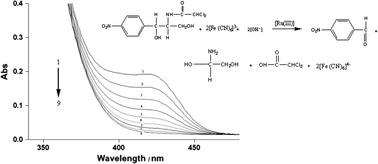

**Electronic supplementary material:**

The online version of this article (doi:10.1007/s00706-014-1208-7) contains supplementary material, which is available to authorized users.

## Introduction

Hexacyanoferrate(III) [HCF(III)] has been widely used to oxidize numerous organic and inorganic compounds in alkaline media. Some authors [1, 2] have suggested that alkaline HCF(III) ion simply acts as an electron-abstracting reagent in redox reactions. However, Speakman and Waters [3] suggested different paths of oxidation of aldehydes, ketones, and nitroparaffins by HCF(III). Singh et al. [4] while discussing the oxidations of formaldehyde, acetone, and ethyl methyl ketone by HCF(III) suggested that the oxidation takes place via an electron transfer process resulting in the formation of a free radical intermediate. HCF(III) is a one-electron oxidant with a redox potential of +0.45 V for the [Fe(CN)_6_]^3−^/[Fe(CN)_6_]^4−^ couple in alkaline medium, leading to its reduction to hexacyanoferrate(II), a stable product [5, 6].

Transition metals are known to catalyze many oxidation–reduction reactions, because they involve multiple oxidation states. In recent years the use of transition metal ions such as ruthenium, osmium, palladium, manganese, chromium, and iridium, either alone or as binary mixtures, as catalysts in various redox processes has attracted considerable interest [7]. Ruthenium(III) acts as a catalyst in the oxidation of many organic and inorganic substrates [8, 9]. Although the mechanism of the catalysis depends on the nature of the substrates, oxidant, and experimental conditions, it has been shown [10] that metal ions acts as catalysts by one of several different paths, such as the formation of complexes with reactants or oxidation of the substrate itself or through the formation of free radicals. Ruthenium(III) catalysis in redox reactions involves different degrees of complexity, owing to the formation of different intermediate complexes and different oxidation states of ruthenium.

Chloramphenicol (2,2-dichloro-*N*-[(1*R*,2*R*)-2-hydroxy-1-(hydroxymethyl)-2-(4-nitrophenyl)ethyl]acetamide, CHP) is a bacteriostatic antimicrobial. Chloramphenicol is effective against a wide variety of Gram-positive and Gram-negative bacteria, including most anaerobic organisms. It is considered a prototypical broad-spectrum antibiotic, alongside the tetracyclines. One important clinical application of chloramphenicol is in the treatment of typhoid. The most serious adverse effect associated with chloramphenicol treatment is bone marrow toxicity. As a result of its extensive usage, chloramphenicol may enter the environment via wastewater effluent and biosolids from sewage treatment plants and via manure and litters from food-producing animal husbandry. The presence and accumulation of chloramphenicol antibiotics in aquatic environments, albeit at low concentrations, may pose threats to the ecosystem and human health by inducing increase and spread of bacteria drug-resistance due to long-term exposure. This necessitates the development of the various advanced oxidation processes for the transformation of chloramphenicol in water [11].

Although some work on oxidation of CHP by various oxidants has been carried out [11, 12] there is a lack of literature on the oxidation of this drug by HCF(III) and its catalysis by ruthenium(III). We have observed that ruthenium(III) in microamounts catalyzes the oxidation of chloramphenicol by HCF(III) in alkaline medium. Such studies are of much significance in understanding the mechanistic profile of chloramphenicol in redox reactions and provide an insight into the interaction of metal ions with the substrate and its mode of action in biological systems. Also, to determine the active species of HCF(III) and ruthenium(III) catalyst, and to resolve the complicity of the reaction, a detailed study of the reaction becomes important. Hence, the present investigation aimed to establish the reactivity of chloramphenicol towards HCF(III) in both uncatalyzed and ruthenium(III)-catalyzed reactions and to arrive at plausible mechanisms.

## Results and discussions

### Stoichiometry and product analysis

Different sets of reaction mixtures containing varying ratios of HCF(III) to CHP in the presence of constant amounts of OH^−^ and NaClO_4_ in an uncatalyzed reaction and with a constant amount of Ru(III) in a catalyzed reaction and at constant ionic strength of 1.10 mol dm^−3^ were allowed to react for about 5 h at 25 °C. The remaining concentration of HCF(III) was assayed by measuring the absorbance at 420 nm. The results indicated 1:2 stoichiometry for both reactions as given in Scheme 1.

**Figure:**
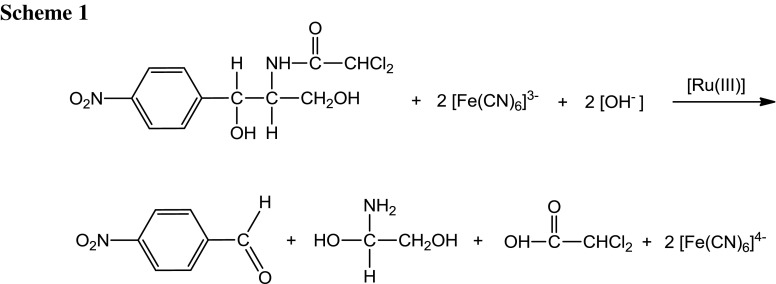

**Fig. 1:**
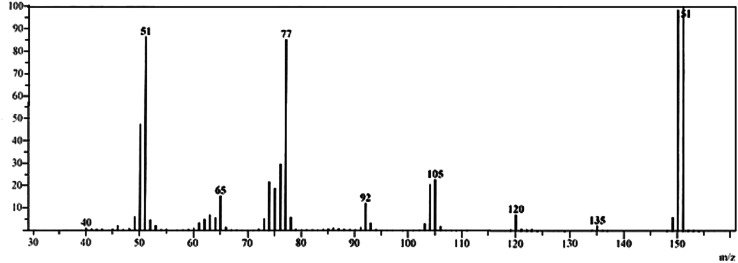
GC–MS spectra of the product *p*-nitrobenzaldehyde showed molecular ion peak and base peak at *m*/*z* = 151

**Fig. 2:**
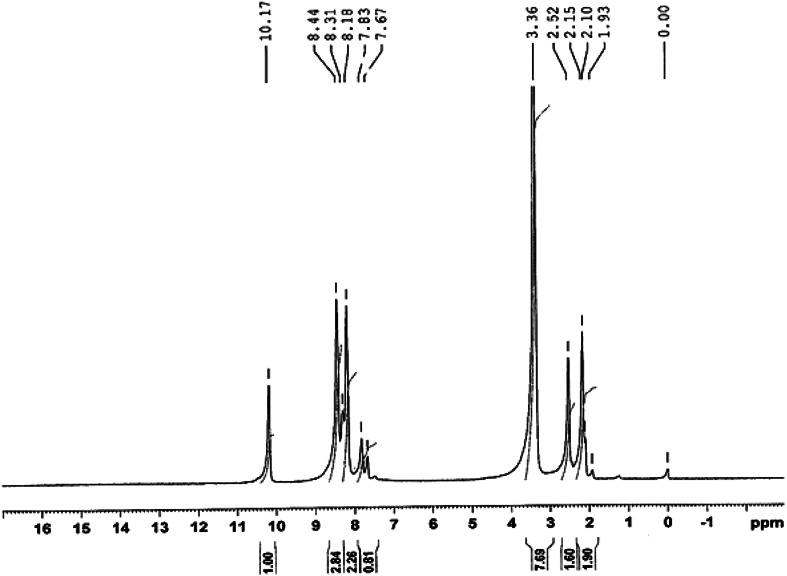
^1^H NMR spectra of *p*-nitrobenzaldehyde, the product of oxidation of chloramphenicol by HCF(III)

After the completion of the reaction, the reaction mixture was acidified, concentrated, and extracted with ether. The reaction product was further recrystallized from aqueous alcohol. The main reaction product was identified as *p*-nitrobenzaldehyde. This was the only organic product obtained in the oxidation which was confirmed by a single spot on thin-layer chromatography and was characterized by spectral investigations. From the IR (Suppl. Fig. 1), GC–MS (Fig. 1), and NMR (Fig. 2) spectra, the main oxidation product was identified as *p*-nitrobenzaldehyde. The IR spectrum showed a C=O stretching band for the aldehyde functional group at 1,709 cm^−1^ and an –NO_2_ stretching band at 1,349 cm^−1^ (Suppl. Fig. 1). The presence of *p*-nitrobenzaldehyde was also confirmed by GC–MS analysis (Fig. 1). The mass spectrum showed a base peak at *m*/*z* = 151 which is consistent with a molecular ion of 151 amu (Fig. 1). All other peaks observed in the GC–MS data can be interpreted in accordance with the structure of *p*-nitrobenzaldehyde. *p*-Nitrobenzaldehyde was also characterized by its ^1^H NMR spectra (Fig. 2, DMSO-*d*_6_). Another product, 2-amino-1,2-ethandiol, was confirmed by GC–MS which showed a molecular ion peak at *m*/*z* = 77. 2-Chloroacetic acid was identified by spot tests [13]. Its reaction products do not undergo further oxidation under the present kinetic conditions.

### Reaction orders

The reaction orders have been determined from the slopes of *k*_U_ or *k*_C_ vs. log(concentration) plots by varying the concentrations of chloramphenicol, OH^−^, or ruthenium(III), in turn, while keeping the others constant.

### Dependence on [HCF(III)]

The oxidant was varied in the absence and presence of catalyst, ruthenium(III), in the concentration range 1.5 × 10^−5^–2.0 × 10^−4 ^mol dm^−3^. The pseudo-first-order rate constants (*k*_U_ and *k*_C_) in both cases were almost constant (Tables 1, 2), indicating first-order dependence with respect to HCF(III) concentration. This was also confirmed by the plots of log(absorbance) vs. time which were linear over three half-lives of the reaction for different initial HCF(III) concentrations.

**Table 1:** Effect of variation of HCF(III), chloramphenicol, and OH^−^ on the oxidation of chloramphenicol by HCF(III) at 25 °C and *I* = 1.10 mol dm^−3^

[HCF] × 10^4^ /mol dm^−3^	[CHP] × 10^3^/mol dm^−3^	[OH^−^]/mol dm^−3^	*k*_U_ × 10^3^/s^−1^
0.15	1.0	0.5	3.48
0.25	1.0	0.5	3.37
0.5	1.0	0.5	3.21
0.7	1.0	0.5	3.13
1.0	1.0	0.5	3.50
2.0	1.0	0.5	3.06
2.0	1.0	0.5	3.06
2.0	2.0	0.5	4.28
2.0	4.0	0.5	5.73
2.0	6.0	0.5	6.81
2.0	8.0	0.5	7.06
2.0	10.0	0.5	7.78
2.0	1.0	0.1	1.05
2.0	1.0	0.3	2.46
2.0	1.0	0.5	3.06
2.0	1.0	0.7	3.53
2.0	1.0	0.9	3.80
2.0	1.0	1.0	4.03

**Table 2:** Effect of variation of HCF(III), chloramphenicol, and OH^−^ on the ruthenium(III)-catalyzed oxidation of chloramphenicol by HCF(III) in aqueous alkaline medium at 25 °C and *I* = 1.10 mol dm^−3^

[HCF] × 10^4^ /mol dm^−3^	[CHP] × 10^3^/mol dm^−3^	[OH^−^]/mol dm^−3^	[Ru(III)] × 10^6^/mol dm^−3^	*k*_T_ × 10^2^/s^−1^	*k*_U_ × 10^3^/s^−1^	*k*_C_ × 10^2^/s^−1^
0.15	1.0	0.5	4.0	2.33	3.48	1.98
0.25	1.0	0.5	4.0	2.45	3.37	2.11
0.5	1.0	0.5	4.0	2.28	3.21	1.95
0.7	1.0	0.5	4.0	2.39	3.13	2.08
1.0	1.0	0.5	4.0	2.70	3.50	2.35
2.0	1.0	0.5	4.0	2.22	3.06	1.90
2.0	1.0	0.5	4.0	2.20	3.06	1.90
2.0	2.0	0.5	4.0	3.07	4.28	2.65
2.0	4.0	0.5	4.0	4.02	5.73	3.45
2.0	6.0	0.5	4.0	4.49	6.81	3.81
2.0	8.0	0.5	4.0	5.23	7.06	4.53
2.0	10.0	0.5	4.0	5.55	7.78	4.78
2.0	1.0	0.1	4.0	0.72	1.05	0.62
2.0	1.0	0.3	4.0	1.45	2.46	1.21
2.0	1.0	0.5	4.0	2.20	3.06	1.90
2.0	1.0	0.7	4.0	2.44	3.53	2.09
2.0	1.0	0.9	4.0	2.79	3.80	2.41
2.0	1.0	1.0	4.0	3.31	4.03	2.91
2.0	1.0	0.5	2.0	1.49	3.06	1.19
2.0	1.0	0.5	4.0	2.20	3.06	1.90
2.0	1.0	0.5	6.0	4.03	3.06	3.73
2.0	1.0	0.5	8.0	5.71	3.06	5.41
2.0	1.0	0.5	10.0	6.84	3.06	6.54
2.0	1.0	0.5	20.0	13.23	3.06	12.93

### Dependence on [substrate]

**Fig. 3:**
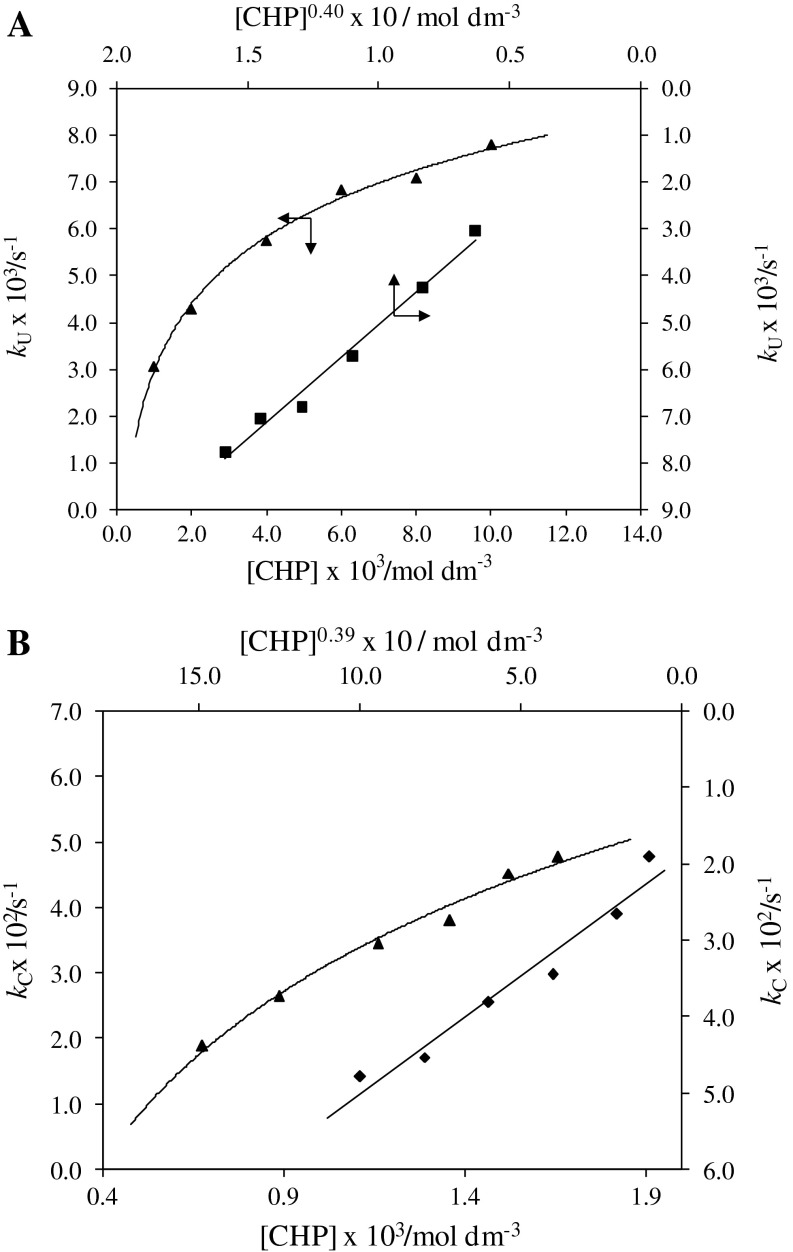
Plots of **a** *k*_U_ vs. [CHP]^0.40^ and *k*_U_ vs. [CHP] (conditions as in Table 1) and **b** *k*_C_ vs.[CHP]^0.39^ and *k*_C_ vs. [CHP] (conditions as in Table 2)

The effect of chloramphenicol was studied for both cases in the concentration range 1.0 × 10^−3^–10.0 × 10^−3^ mol dm^−3^ at a constant concentration of HCF(III), OH^−^, and a constant ionic strength of 1.10 mol dm^−3^ in the uncatalyzed reaction and with a constant concentration of Ru(III) in the catalyzed reaction. In both cases, at constant temperature, the *k*_U_ and *k*_C_ values increased with the increase in [CHP] (Tables 1, 2). The order with respect to [CHP] was less than unity. This was also confirmed by the plots of *k*_U_ vs. [CHP]^0.40^ and *k*_C_ vs. [CHP]^0.39^ which were linear, unlike the direct plot of *k*_U_ vs. [CHP] and *k*_C_ vs. [CHP] (Fig. 3).

### Dependence on [alkali]

The effect of alkali was studied for both cases in the concentration range 0.10–1.0 mol dm^−3^ at constant concentrations of HCF(III), CHP, and ionic strength in the uncatalyzed reaction and with a constant concentration of Ru(III) in the catalyzed reaction. The rate constants increased with the increase in [alkali] (Tables 1, 2) and the order was found to be less than unity, i.e., 0.57 in the uncatalyzed reaction and 0.64 in the catalyzed reaction.

### Dependence on [ruthenium(III)]

**Fig. 4:**
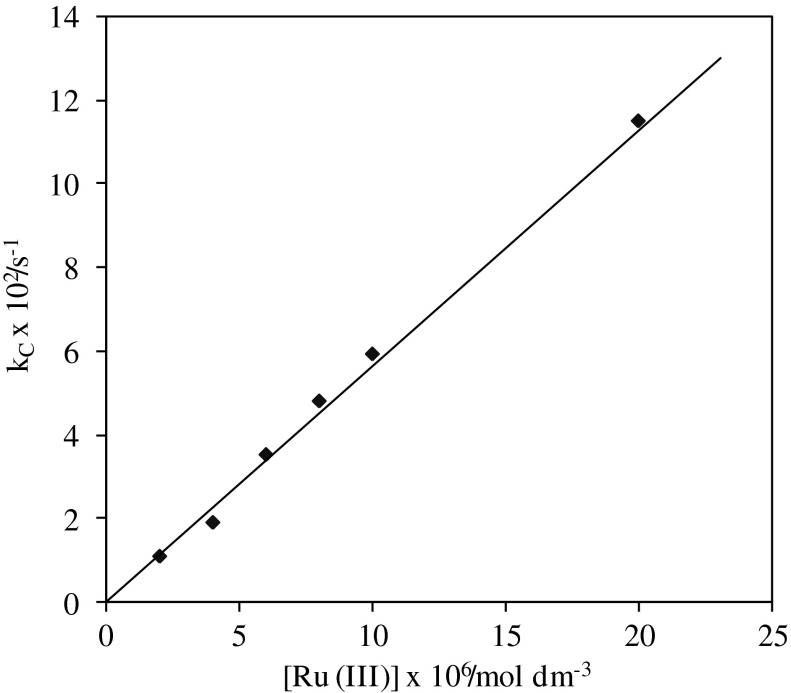
Unit order plot of [Ru(III)] vs. *k*_C_

Ruthenium(III) concentration was varied from 2.0 × 10^−6^ to 2.0 × 10^−5 ^mol dm^−3^, at constant concentrations of HCF(III), CHP, and alkali, and at constant ionic strength. As the concentration of ruthenium(III) increases the rate of reaction also increases (Table 2). The order with respect to concentration of Ru(III) was found to be unity (Fig. 4).

### Effect of ionic strength (*I*) and dielectric constant (*D*)

In the absence and in the presence of catalyst, constant concentrations of reactants and with other conditions constant, the ionic strength was varied by varying NaClO_4_ concentration between 0.6, 0.8, 0.9, 1.1, and 1.4 mol dm^−3^. The rate was found to increase with increase in ionic strength. A plot of log*k*_U_ or *k*_C_ vs. *I* ^1/2^ was linear with positive slope (Suppl. Fig. 2). The effect of dielectric constant was studied by varying the *t*-butyl alcohol–water volume fractions from 0 to 30. It was found that as the volume fractions of *t*-butyl alcohol increased in the reaction medium, the rate of reaction increased in the absence and presence of catalyst (Suppl. Fig. 3). The plot of log*k*_U_ or *k*_C_ vs. 1/*D* was linear with a positive slope.

### Effect of initially added products

The initially added products *p*-nitrobenzaldehyde and hexacyanoferrate(II) did not have any significant effect on the rate of reaction in the absence and presence of catalyst.

### Test for free radicals (polymerization study)

For both the uncatalyzed and catalyzed reactions, the intervention of the free radicals in the reaction was examined as follows: the reaction mixture, to which a known quantity of acrylonitrile monomer (scavenger) had been added initially, was kept for 2 h in an inert atmosphere. On diluting the reaction mixture with methanol, a white precipitate was formed, indicating the intervention of free radicals in the reactions. The blank experiments of either [Fe(CN)_6_]^3−^ or CHP alone with acrylonitrile did not induce any polymerization under the same conditions as those induced for the reaction mixture. Initially added acrylonitrile decreases the rate also indicating the free radical intervention [14].

### Effect of temperature

The kinetics were studied at four different temperatures 15, 25, 35, and 45 °C under varying concentrations of chloramphenicol and alkali keeping the other conditions constant for the uncatalyzed reaction. The rate constants (*k*_1_) of the slow step of Scheme 2 were obtained from the intercepts of the plots of 1/*k*_U_ vs. 1/[CHP] at the four different temperatures. The values are given in Table 3. The energy of activation for the rate-determining step was obtained by the least-squares method of the plot of log*k*_1_ vs. 1/*T*, and the other activation parameters are calculated and are given in Table 3. For the catalyzed reaction the influence of temperature on the rate of reaction was also studied at 15, 25, 35, and 45 °C. The rate constants (*k*_2_) of the slow step of Scheme 3 were obtained from the intercepts of the plots of [Ru(III)]/*k*_C_ vs. 1/[CHP] at the four different temperatures. The values are given in Table 4. The energy of activation for the rate determining step was obtained by the least-squares method of the plot of log*k*_2_ vs. 1/*T* and other activation parameters calculated for the reaction are presented in Table 4.

**Figure:**
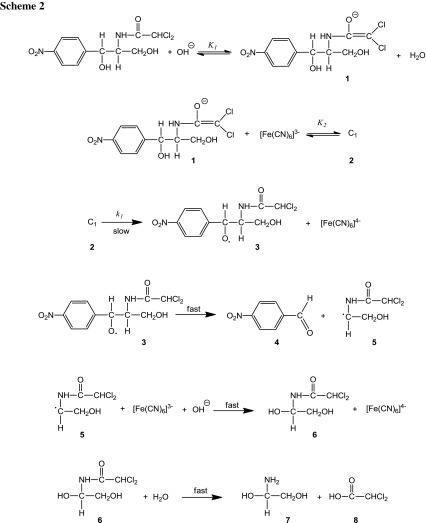

**Table 3:** Activation parameters and thermodynamic quantities for the oxidation of CHP by HCF(III) in alkaline medium with respect to the slow step of Scheme 2

Effect of temperature and activation parameters			
Temperature/K	*k*_1_ × 10^2^/s^−1^	Parameters	Values
288	0.44	*E*_a_/kJ mol^−1^	53 ± 3
298	0.88	Δ*H* ^#^/kJ mol^−1^	51 ± 3
308	1.77	Δ*S* ^#^/J K^−1^ mol^−1^	−114 ± 10
318	3.54	Δ*G* ^#^/kJ mol^−1^	84 ± 3
		log*A*	7.2 ± 0.3

Effect of temperature on first and second equilibrium steps of Scheme 2			
Temperature/K	*K*_1_ × 10^1^/dm^3^ mol^−1^	*K*_2_ × 10^3^/dm^3^ mol^−1^	
288	0.38	3.30	
298	0.71	2.07	
308	1.32	1.37	
318	2.68	0.98	

Thermodynamic quantities with respect to *K*_1_ and *K*_2_			
Thermodynamic quantities	Values from *K*_1_	Values from *K*_2_	
Δ*H*/kJ mol^−1^	49	−30.8	
Δ*S*/J K^−1^ mol^−1^	162	−40.4	
Δ*G*_298_/kJ mol^−1^	0.84	−18.92	

[HCF] = 2.0 × 10^−4^ mol dm^−3^, [CHP] = 1.0 × 10^−3^ mol dm^−3^, [OH^−^] = 0.5 mol dm^−3^, *I* = 1.10 mol dm^−3^

**Figure:**
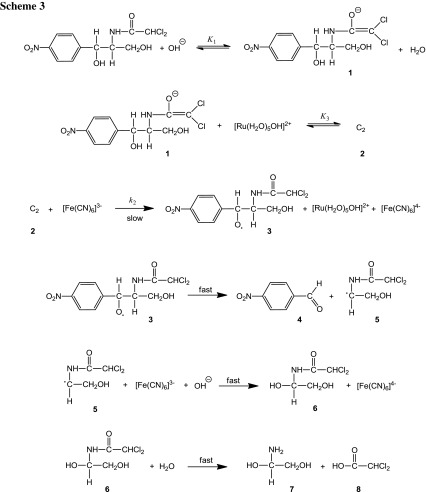

**Table 4:** Activation parameters and thermodynamic quantities for the ruthenium(III)-catalyzed oxidation of CHP by HCF(III) in aqueous alkaline medium with respect to the slow step of Scheme 3

Effect of temperature and activation parameters			
Temperature/K	*k*_2_ × 10^−5^/dm^3 ^mol^−1 ^s^−1^	Parameters	Values
288	0.65	*E*_a_/kJ mol^−1^	48 ± 3
298	1.30	Δ*H* ^#^/kJ mol^−1^	46 ± 3
308	2.60	Δ*S* ^#^/J K^−1^ mol^−1^	−123 ± 10
318	5.20	Δ*G* ^#^/kJ mol^−1^	83 ± 3
		log*A*	12.7 ± 0.3

Effect of temperature on first and second equilibrium steps in Scheme 3			
Temperature/K	*K*_1_ × 10^1^/dm^3^ mol^−1^	*K*_2_ × 10^3^/dm^3^ mol^−1^	
288	0.36	3.50	
298	0.57	2.45	
308	1.15	1.53	
318	2.90	0.98	

Thermodynamic quantities with respect to *K*_1_ and *K*_2_			
Thermodynamic quantities	Values from *K*_1_	Values from *K*_2_	
Δ*H*/kJ mol^−1^	52.62	−33	
Δ*S*/J K^−1^ mol^−1^	173.15	−46	
Δ*G*_298_/kJ mol^−1^	1.38	−19	

### Catalytic activity

Molelwyn-Hughes [15] pointed out that in the presence of catalyst, the uncatalyzed and catalyzed reactions proceed simultaneously, so that

1 $$ k_{\text{T}} = k_{\text{U}} + K_{\text{C}} [{\text{Ru}}({\text{III}})]^{x} $$

where *k*_T_ is the total rate constant, *k*_U_ the pseudo-first-order rate constant for the uncatalyzed path, *K*_C_ the catalytic constant, and *x* the order of the reaction with respect to catalyst. In the present investigations the *x* value for the standard run was found to be unity. Then, the value of *K*_C_ is calculated using the equation

2 $$ K_{\text{C}} = \frac{{k_{\text{T}} - k_{\text{U}} }}{{[{\text{Ru}}({\text{III}})]^{x} }} = \frac{{k_{\text{C}} }}{{[{\text{Ru}}({\text{III}})]}}\;({\text{where}}\;k_{\text{T}} - k_{\text{U}} = k_{\text{C}} ) $$

The values of *K*_C_ were evaluated for Ru(III) catalyst at four different temperatures (Table 5). Further, plots of log*K*_C_ vs. 1/*T* were linear and the values of activation parameters with reference to catalyst were computed. These results are summarized in Table 5.

**Table 5:** Values of catalytic constant (*K*_C_) at different temperatures and activation parameters calculated using *K*_C_ values

Temperature/K	*K*_C_ × 10^−3^	Parameters	Values
288	2.37	*E*_a_/kJ mol^−1^	53 ± 3
298	4.75	Δ*H* ^#^/kJ mol^−1^	50 ± 3
308	9.50	Δ*S* ^#^/J K^−1^ mol^−1^	−5.8 ± 0.3
318	19.00	Δ*G* ^#^/kJ mol^−1^	52 ± 3
		log*A*	12.9 ± 0.4

[HCF] = 2.0 × 10^−4^ mol dm^−3^, [CHP] = 1.0 × 10^−3^ mol dm^−3^, [OH^−^] = 0.5 mol dm^−3^, [Ru(III)] = 4.0 × 10^−6^, *I* = 1.10 mol dm^−3^

### Mechanism of uncatalyzed reaction

The variation of the concentrations of the oxidant HCF(III), substrate (CHP), and alkali, while keeping others constant, showed that the reaction is first order in oxidant, alkali, and of fractional order in substrate concentrations (Table 1). The reaction between chloramphenicol and Fe[(CN)_6_]^3−^ has a stoichiometry of 1:2. On the basis of the experimental results, a mechanism can be proposed for which all the observed orders in each constituent, i.e., [oxidant], [reductant], and [OH^−^], may be well accounted for. Oxidation of chloramphenicol by HCF(III) in NaOH media is a non-complementary reaction with oxidant undergoing equivalent changes.

In the present study, alkali combines first with chloramphenicol to give the anionic form of chloramphenicol (**1**) in a pre-equilibrium step, which is also supported by the observed fractional order in [OH^−^] and [CHP]. The HCF(III) species reacts with the anionic form of chloramphenicol to give a complex C_1_ (**2**), which decomposes in a slow step to give a free radical **3** derived from the chloramphenicol anion and Fe[(CN)_6_]^4−^. This free radical in a subsequent fast step decomposes to give *p*-nitrobenzaldehyde (**4**) and another free radical **5**. In the next fast step free radical **5** reacts with another mole of HCF in the presence of OH^−^ to form an intermediate 2,2-dichloro-*N*-(1,2-dihydroxyethyl)acetamide (**6**). In the further fast steps, **6** undergoes hydrolysis to give the final products 2-amino-1,2-ethandiol (**7**) and dichloroacetic acid (**8**) as given in Scheme 2.

Since Scheme 2 is in accordance with the generally well-accepted principle of non-complementary oxidations taking place in a sequence of one-electron steps, the reaction between the substrate and oxidant would afford a radical intermediate. A free radical scavenging experiment revealed such a possibility. Spectroscopic evidence for the complex formation between oxidant and substrate was obtained from UV–Vis spectra of HCF (2.0 × 10^−4^ mol dm^−3^), CHP (1.0 × 10^−3^ mol dm^−3^), [OH^−^] (0.5 mol dm^−3^), and a mixture of both. A bathochromic shift of about 8 nm from 260 to 268 nm in the spectra of HCF to mixture of HCF and CHP was observed. A Michaelis–Menten plot proved the complex formation between oxidant and substrate, which explains the fractional order in [CHP].

Scheme 2 leads to the rate law Eq. (3):

3 $$ {\text{Rate}} = \frac{{ - {\text{d}}[{\text{Fe}}({\text{CN}})_{6}^{3 - } ]}}{{{\text{d}}t}} = \frac{{k_{1} K_{1} K_{2} [{\text{CHP}}][{\text{OH}}^{ - } ][{\text{Fe}}({\text{CN}})_{6}^{3 - } ]}}{{1 + K_{1} [{\text{OH}}^{ - } ] + K_{1} K_{2} [{\text{CHP}}][{\text{OH}}^{ - } ]}} $$

4 $$ k_{\text{U}} = \frac{\text{Rate}}{{[{\text{Fe}}({\text{CN}})_{6}^{3 - } ]}} = \frac{{k_{1} K_{1} K_{2} [{\text{CHP}}][{\text{OH}}^{ - } ]}}{{1 + K_{1} [{\text{OH}}^{ - } ] + K_{1} K_{2} [{\text{CHP}}][{\text{OH}}^{ - } ]}} $$

Equation (4) can be rearranged to the following form, which is suitable for verification:

5 $$ \frac{1}{{k_{\text{C}} }} = \frac{1}{{k_{1} K_{1} K_{2} [{\text{CHP}}][{\text{OH}}^{ - } ]}} + \frac{1}{{K_{1} K_{2} [{\text{CHP}}]}} + \frac{1}{{k_{1} }} $$

**Fig. 5:**
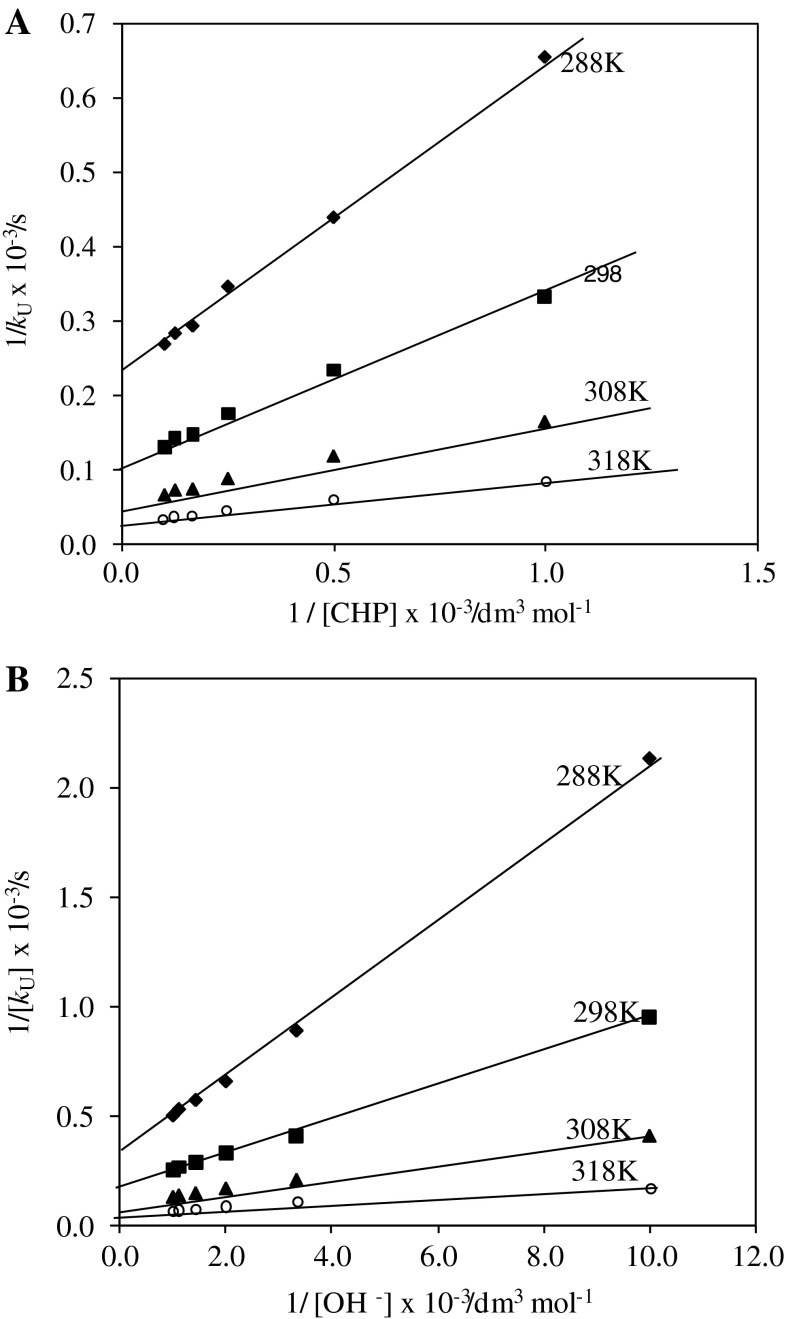
Verification of rate law Eq. (4) for the oxidation of chloramphenicol by HCF(III). Plots of **a** 1/*k*_U_ vs. 1/[CHP]; **b** 1/*k*_U_ vs. 1/[OH^−^] at four different temperatures (conditions as in Table 1)

According to Eq. (5), other conditions being constant, plots of 1/*k*_U_ vs. 1/[CHP], 1/*k*_U_ vs. 1/[OH^−^] should be linear and are found to be so (Fig. 5). The slopes and intercepts of such plots lead to the values of *K*_1_, *K*_2_, and *k*_1_ (Table 3).

The effect of ionic strength and dielectric constant of medium on the rate explains qualitatively the reaction between ions having the same charge, as seen in Scheme 2. The thermodynamic quantities for the different equilibrium steps in Scheme 2 can be evaluated as follows. The [CHP] and [OH^−^] (Table 1) were varied at four different temperatures. The plots of 1/*k*_U_ vs. 1/[CHP], 1/*k*_U_ vs. 1/[OH^−^] should be linear and are found to be so (Fig. 5). From the slopes and intercepts, the values of *K*_1_, *K*_2_ were calculated at different temperatures (Table 3). A van’t Hoff plot was drawn for the variation of *K*_1_ and *K*_2_ with temperature (log*K*_1_ vs. 1/*T* and log*K*_2_ vs. 1/*T*). The values of enthalpy of reaction Δ*H*, entropy of reaction Δ*S*, and free energy of reaction Δ*G* were calculated for the first and second equilibrium steps. These values are given in Table 3. A comparison of the Δ*H* value (49 kJ mol^−1^) from *K*_1_ of the first step with that of Δ*H* ^#^ (51 kJ mol^−1^) obtained for the rate-determining step shows that the reaction before the rate-determining step is fairly fast as it involves low activation energy [16]. A high negative value of Δ*S* ^#^ (−114 J K^−1^ mol^−1^) suggests that intermediate complex (C_1_) is more ordered than the reactants [17].

### Mechanism for ruthenium(III)-catalyzed reaction

The variation of the concentrations of the oxidant [Fe(CN)_6_]^3−^, substrate (chloramphenicol), Ru(III), and alkali, while keeping others constant, showed that the reaction is first order in oxidant and in Ru(III) and of fractional order in alkali and substrate concentrations (Table 2). The reaction between chloramphenicol and [Fe(CN)_6_]^3−^ in NaOH in the presence of Ru(III) has a stoichiometry of 1:2. On the basis of the experimental results, a mechanism can be proposed for which all the observed orders in each constituent, i.e., [oxidant], [reductant], catalyst, and [OH^−^], may be well accounted for. Oxidation of chloramphenicol by HCF(III) in NaOH media is a non-complementary reaction [18].

Ruthenium(III) chloride acts as an efficient catalyst in many redox reactions, particularly in an alkaline medium [19]. In the present study it is quite probable that the [Ru(H_2_O)_5_OH]^2+^ species might assume the general form [Ru(III)(OH)_*x*_]^3−*x*^. The *x* value must always be less than 6 because there are no definite reports of any hexahydroxy ruthenium species. The remainder of the coordination sphere would be filled by water molecules. Hence, under the conditions employed, e.g., [OH^−^] ≫ [Ru(III)], ruthenium(III) is mostly present [20] as hydroxylated species, [Ru(H_2_O)_5_OH]^2+^.

In earlier reports of Ru(III)-catalyzed oxidation, it was observed [21] that, if there exists a fractional order dependence with respect to [substrate] and [Ru(III)], and with respect to [oxidant], it leads to the formation of Ru(III)–substrate complex. This complex is further oxidized by the oxidant to Ru(III)–substrate complex followed by the rapid redox decomposition with regeneration of Ru(III) catalyst. In another case [22], if the process involves a zero-order dependence with respect to [oxidant], first order with respect to [Ru(III)], and a fractional order with respect to [substrate], it leads to the formation of Ru(III)–substrate complex and further cleaves to Ru(I) species which is rapidly oxidized by the oxidant to regenerate Ru(III) catalyst.

The results indicate that the alkali combines first with chloramphenicol to give the anionic form of chloramphenicol in a pre-equilibrium step, which is also supported by the observed fractional order in [OH^−^] and [CHP]. The ruthenium(III) species then reacts with the anionic form of chloramphenicol to give a complex C_2_ (**2**), which decomposes in the presence of the HCF(III) species in a slow step to give a free radical **3** derived from the chloramphenicol anion and Fe[(CN)_6_]^4−^ with regeneration of the catalyst, ruthenium(III). *K*_3_ is the equilibrium constant for the equilibrium binding of chloramphenicol to ruthenium(III). This free radical **3** in a subsequent fast step decomposes to give *p*-nitrobenzaldehyde (**4**) and another free radical **5**. In the next fast step, free radical **5** reacts with another mole of HCF in the presence of OH^−^ to give an intermediate 2,2-dichloro-*N*-(1,2-dihydroxyethyl)acetamide (**6**). In a further fast step **6** undergoes hydrolysis to give the final products 2-amino-1,2-ethandiol (**7**) and dichloroacetic acid (**8**) as given in Scheme 3.

Spectroscopic evidence for the complex formation between Ru(III) and CHP was obtained from UV–Vis spectra of [CHP] (1.0 × 10^−3 ^mol dm^−3^), [Ru(III)] (4.0 × 10^−6^ mol dm^−3^), [OH^−^] (0.5 mol dm^−3^), and a mixture of both. A bathochromic shift of about 5 nm from 278 to 283 nm in the spectra of Ru(III) to mixture of Ru(III) and CHP was observed. Michaelis–Menten plot proved the complex formation between a catalyst and substrate, which explains the fractional order in [CHP].

From Scheme 3, the rate law Eq. (6) can be derived:

6 $$ \frac{\text{Rate}}{{[{\text{Fe}}({\text{CN}})_{6}^{3 - } ]}} = k_{\text{C}} = \frac{{k_{2} K_{1} K_{3} [{\text{CHP}}][{\text{OH}}^{ - } ][{\text{Ru}}({\text{H}}_{2} {\text{O}})_{5} {\text{OH}}]^{2 + } }}{{1 + K_{1} [{\text{OH}}^{ - } ] + K_{1} K_{3} [{\text{CHP}}][{\text{OH}}^{ - } ]}} $$

Equation (6) can be rearranged to Eq. (7) and is used for verification:

7 $$ \frac{{[{\text{Ru}}({\text{III}})]}}{{k_{\text{C}} }} = \frac{1}{{k_{2} K_{1} K_{3} [{\text{CHP}}][{\text{OH}}^{ - } ]}} + \frac{1}{{K_{2} K_{3} [{\text{CHP}}]}} + \frac{1}{{k_{2} }} $$

**Fig. 6:**
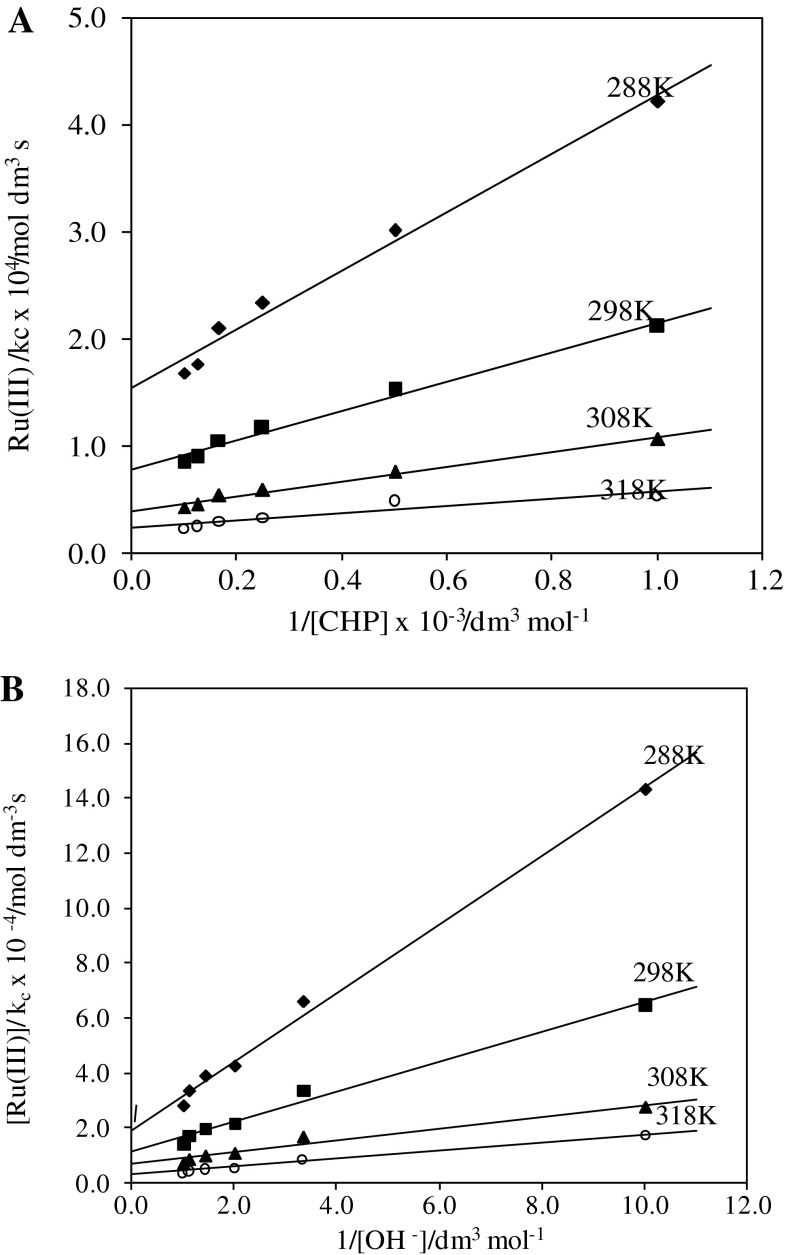
Verification of rate law Eq. (6) for the Ru(III)-catalyzed oxidation of chloramphenicol by hexacyanoferrate(III). Plots of **a** [Ru(III)]/*k*_C_ vs. 1/[CHP], **b** [Ru(III)]/*k*_C_ vs. 1/[OH^−^] at four different temperatures (conditions as in Table 2)

According to Eq. (7), other conditions being constant, plots of [Ru(III)]/*k*_C_ vs. 1/[CHP] and 1/[OH^−^] should be linear and are found to be so (Fig. 6). The slopes and intercepts of such plots lead to the values of *K*_1_, *K*_3_, and *k*_2_ (Table 4).

The effect of ionic strength and dielectric constant of the medium on the rate qualitatively explains the reaction between ions having the same charge, as seen in Scheme 3. The thermodynamic quantities for the different equilibrium steps in Scheme 3 can be evaluated as follows. The [CHP] and [OH^−^] (Table 2) were varied at four different temperatures. The plots of [Ru(III)]/*k*_C_ vs. 1/[CHP], [Ru(III)]/*k*_C_ vs. 1/[OH^−^] should be linear and are found to be so (Fig. 6). From the slopes and intercepts, the values of *K*_1_ and *K*_3_ were calculated at different temperature (Table 4b). A van’t Hoff plot was drawn for the variation of *K*_1_ and *K*_3_ with temperature (log*K*_1_ vs. 1/*T* and log*K*_3_ vs. 1/*T*). The values of enthalpy of reaction Δ*H*, entropy of reaction Δ*S*, and free energy of reaction Δ*G* were calculated for the first and second equilibrium steps. These values are given in Table 4. The negative value of Δ*S* ^#^ (−123 J K^−1^ mol^−1^) suggests that the intermediate complex (C_2_) is more ordered than the reactants [17]. The observed modest enthalpy of activation and higher rate constants for the slow step indicate that the oxidation presumably occurred via an inner sphere mechanism. This conclusion is supported by earlier observations [23, 24]. The activation parameters evaluated for the catalyzed and uncatalyzed reactions explain the catalytic effect on the reaction. The catalyst Ru(III) forms the complex (C_2_) with substrate, which enhances the reducing property of the substrate relative to that without catalyst. Further, the catalyst Ru(III) modifies the reaction path by lowering the energy of activation.

It is also interesting to note that the transient species involved in both uncatalyzed and Ru(III)-catalyzed reactions is different but leads to formation of the same products. The uncatalyzed reaction in alkaline medium has been shown to proceed via a HCF–CHP complex which decomposes slowly in a rate-determining step to give the products via free radicals in the further steps, whereas, in the catalyzed reaction, it has been shown to proceed via a Ru(III)–CHP complex which further reacts with 1 mole of HCF in the rate-determining step to give the products via free radicals in the further steps. Since in both cases HCF and CHP were involved, the products obtained were the same.

## Conclusions

A comparative study of uncatalyzed and Ru(III)-catalyzed oxidation of chloramphenicol by HCF(III) in alkaline medium was performed. The active species of Ru(III) is found to be [Ru(H_2_O)_5_OH]^2+^. The reaction rates are about tenfold faster than those of the uncatalyzed reaction. It becomes apparent that, in carrying out this reaction, the role of reaction medium is crucial. Activation parameters were evaluated for both catalyzed and uncatalyzed reactions. Catalytic constants and activation parameters with reference to the catalyst were also computed. The overall sequence described here is consistent with all experimental findings including the product, spectral, mechanistic, and kinetic studies.

## Experimental

All chemicals were of analytical reagent grade and millipore water was used throughout the work. The solution of chloramphenicol (SISCO CHEM) was prepared by dissolving known amounts of the samples in millipore water. The purity of the sample was checked by their melting point (150 °C). Solutions of chloramphenicol were always freshly prepared before use. A stock solution of the oxidant, HCF(III), was prepared by dissolving K_3_Fe(CN)_6_ (SISCO CHEM) in millipore water and standardizing the solution iodometrically [25]. The ruthenium(III) solution was prepared by dissolving RuCl_3_ (s. d. fine) in HCl (0.20 mol dm^−3^) and Hg was added to the Ru(III) stock solution to reduce any Ru(IV) formed during the preparation of the Ru(III) stock solution, and was set aside for 24 h. The Ru(III) concentration was assayed by EDTA titration [26]. Hexacyanoferrate(II) solution was prepared by dissolving a known amount of K_4_Fe(CN)_6_ (s. d. fine) in water. NaOH (Merck) and NaClO_4_ (BDH) were used to provide the required alkalinity and to maintain ionic strength, respectively.

For kinetic measurements, a Peltier Accessory (temperature control) attached to a Varian CARY 50 Bio UV–Vis spectrophotometer (Varian, Victoria-3170, Australia) was used. For product analysis, the QP-2010S Shimadzu GC–MS system, Nicolet 5700-FT-IR spectrometer (Thermo, USA), and Bruker 300 MHz ^1^H NMR spectrophotometer (Bruker, Switzerland) were used.

### Procedure and kinetic measurements

The oxidation of chloramphenicol by HCF(III) was followed under pseudo-first-order conditions where [CHP] > [HCF(III)] in both the uncatalyzed and catalyzed reactions at 25.0 ± 0.1 °C, unless otherwise specified. In the absence of catalyst, the reaction was initiated by mixing HCF(III) with the CHP solution, which also contained the required concentrations of NaClO_4_ and NaOH. The reaction in the presence of the Ru(III) catalyst was initiated by mixing HCF(III) with the CHP solution which also contained the required concentrations of NaClO_4_, NaOH, and Ru(III) catalyst. The progress of the reaction was followed by measuring absorbance of Fe[(CN)_6_]^3−^ in the reaction mixture at 420 nm in a 1-cm cell placed in the cell compartment of an Varian carry 50 Bio UV–Vis spectrophotometer. Application of Beer’s law under the reaction conditions had been verified at 420 nm (*ε* = 1,070 ± 10 dm^3^ mol^−1^ cm^−1^) [27]. In uncatalyzed and catalyzed cases, the kinetics was followed to more than 90 % completion of the reaction and good first-order kinetics were observed. During the kinetic studies it was observed that under the present experimental conditions in the absence of catalyst ruthenium(III), the oxidation of chloramphenicol by Fe[(CN)_6_]^3−^ occurs very slowly, but in a measurable quantity. Hence, during the calculation of pseudo-first-order rate constants, *k*_C_, in the presence of catalyst, the uncatalyzed rate has also been taken into account. Therefore in each ruthenium(III)-catalyzed kinetic run, a parallel kinetic run under similar conditions in the absence of ruthenium(III) was also carried out. In both cases the pseudo-first-order rate constants (*k*_U_ and *k*_C_) were obtained from the plots of log(absorbance) vs. time. The pseudo-first-order plots were linear over three half-lives. Thus, the total rate constant (*k*_T_) is equal to the sum of the rate constants in the absence (*k*_U_) and in the presence *k*_C_ of catalyst, i.e.,

$$ \begin{gathered} k_{\text{T}} = k_{\text{U}} + k_{\text{C}} \hfill \\ k_{\text{C}} = k_{\text{T}} {-}k_{\text{U}} \hfill \\ \end{gathered} $$

The rate constants *k*_U_ and *k*_C_ values are shown in Tables 1 and 2. The spectral changes during the chemical reaction for the standard condition at 25 °C are shown in Suppl. Fig. 4. It is evident from the figure that the concentration of HCF(III) decreases at 420 nm. It was also observed that there was almost no interference from other species in the reaction mixture at this wavelength. Similar results were obtained for the degradation of CHP by measuring COD values at different time.

### Electronic supplementary material

Below is the link to the electronic supplementary material.

Supplementary material 1 (DOCX 165 kb)

## Appendix

### Derivation of rate law for uncatalyzed reaction

According to Scheme 2

8 $$ {\text{Rate}} = - \frac{{{\text{d}}[{\text{Fe}}({\text{CN}})_{6}^{3 - } ]}}{{{\text{d}}t}} = k_{1} [{\text{complex}}] $$

9 $$ {\text{Rate}}  = k_{1} K_{1} K_{2} \left[ {\text{CHP}} \right]_{\rm f} \left[ {{\text{OH}}^{ - } } \right]_{\rm f} \left[ {{\text{Fe}}\left( {\text{CN}} \right)_{6}^{3 - } } \right]_{\rm f} $$

The total concentration of [CHP]_T_ is given by

10 $$ \left[ {\text{CHP}} \right]_{\rm T} = \left[ {\text{CHP}} \right]_{\rm f} + \left[ {\text{anionic  form  of  CHP}} \right] + \left[ C \right] $$

where* T* and* f* refer to total and free concentrations

$$ \begin{gathered} = \left[ {\text{CHP}} \right]_{\rm f} + K_{1} \left[ {\text{CHP}} \right]\left[ {{\text{OH}}^{ - } } \right] + K_{2} \left[ {{\text{anionic  from}}\,\,{\text{of  CHP}}({\text{I}})} \right]\left[ {{\text{Fe}}\left( {\text{CN}} \right)_{6}^{3 - } } \right] \hfill \\ = \left[ {\text{CHP}} \right]_{\rm f} + K_{1} \left[ {\text{CHP}} \right]\left[ {{\text{OH}}^{ - } } \right] + K_{1} K_{2} \left[ {{\text{anionic  from}}\,\,{\text{of  CHP}}({\text{I}})} \right]\left[ {{\text{OH}}^{ - } } \right]\left[ {{\text{Fe}}\left( {\text{CN}} \right)_{6}^{3 - } } \right] \hfill \\ = \left[ {\text{CHP}} \right]_{\rm f} \left[ {1 + K_{1} \left[ {{\text{OH}}^{ - } } \right] + K_{1} K_{2} \left[ {{\text{OH}}^{ - } } \right]} \right.\left[ {{\text{Fe}}\left( {\text{CN}} \right)_{6}^{3 - } } \right] \hfill \\ \left[ {\text{CHP}} \right]_{\rm f} = \frac{{\left[ {\text{CHP}} \right]_{\rm T} }}{{\left[ {1 + \left[ {{\text{OH}}^{ - } } \right] + K_{1} K_{2} \left[ {{\text{OH}}^{ - } } \right]{\text{Fe}}\left( {\text{CN}} \right)_{6}^{3 - } } \right].}} \hfill \\ \end{gathered} $$

In view of the low concentration of [OH^−^], the numerator term *K*_1_ *K*_2_[OH][Fe(CN)_6_^3−^] in the above equation is neglected:

11 $$ \left[ {\text{CHP}} \right]_{\rm f} = \frac{{\left[ {\text{CHP}} \right]_{\rm T} }}{{1 + K_{1} \left[ {{\text{OH}}^{ - } } \right]}} $$

Similarly,

$$ \left[ {{\text{OH}}^{ - } } \right]_{\rm T} = \left[ {{\text{OH}}^{ - } } \right] + \left[ {\text{anionic  from  of  CHP}} \right] $$

$$ \left[ {{\text{OH}}^{ - } } \right]_{\rm f} = \frac{{\left[ {{\text{OH}}^{ - } } \right]_{\rm T} }}{{1 + K_{1} \left[ {\text{CHP}} \right]}} $$

In view of the low concentration of [CHP], the numerator term 1 + *K*_1_ [CHP] in the above equation is neglected.

Therefore the total concentration of OH^−^ is given by

12 $$ \left[ {{\text{OH}}^{ - } } \right]_{\rm f} = \left[ {{\text{OH}}^{ - } } \right]_{\rm T} $$

Similarly,

$$ \left[ {{\text{Fe}}\left( {\text{CN}} \right)_{6}^{3 - } } \right]_{\rm T} = \left[ {{\text{Fe}}\left( {\text{CN}} \right)_{6}^{3 - } } \right]_{\rm f} + C $$

13 $$ \left[ {{\text{Fe}}\left( {\text{CN}} \right)_{6}^{3 - } } \right]_{\rm f} = \frac{{\left[ {{\text{Fe}}\left( {\text{CN}} \right)_{6}^{3 - } } \right]_{\rm T} }}{{\left( {1 + K_{1} K_{2} \left[ {\text{CHP}} \right]\left[ {{\text{OH}}^{ - } } \right]} \right)}} $$

Substituting Eqs. (11), (12), and (13) into Eq. (9) and omitting *t* and *f* we get

$$ {\text{Rate}} = \frac{{k_{1} K_{1} K_{2} \left[ {\text{CHP}} \right]\left[ {{\text{OH}}^{ - } } \right]\left[ {{\text{Fe}}\left( {\text{CN}} \right)_{6}^{3 - } } \right]}}{{\left( {1 + K_{1} \left[ {{\text{OH}}^{ - } } \right]} \right)\left( {1 + K_{1} K_{2} \left[ {\text{CHP}} \right]\left[ {{\text{OH}}^{ - } } \right]} \right)}} $$

14 $$ {\text{Rate}} = \frac{{k_{1} K_{1} K_{2} \left[ {\text{CHP}} \right]\left[ {{\text{OH}}^{ - } } \right]\left[ {{\text{Fe}}\left( {\text{CN}} \right)_{6}^{3 - } } \right]}}{{\left( {1 + K_{1} K_{2} \left[ {\text{CHP}} \right]\left[ {{\text{OH}}^{ - } } \right]} \right) + K_{1} \left[ {{\text{OH}}^{ - } } \right]K_{1}^{2} K_{2} \left[ {{\text{OH}}^{ - } } \right]^{2} \left[ {\text{CHP}} \right]}} $$

The term *K*_1_^2^ *K*_2_[CHP][OH^−^] in the denominator of Eq. (14) is negligibly small compared to unity in view of the low concentration of substrate [CHP] used. Therefore Eq. (14) can be written as

15 $$ k_{\text{U}} = \frac{\text{Rate}}{{\left[ {{\text{Fe}}\left( {\text{CN}} \right)_{6}^{3 - } } \right]}} = \frac{{k_{1} K_{1} K_{2} \left[ {\text{CHP}} \right]\left[ {{\text{OH}}^{ - } } \right]}}{{1 + K_{1} \left[ {{\text{OH}}^{ - } } \right] + K_{1} K_{2} \left[ {\text{CHP}} \right]\left[ {{\text{OH}}^{ - } } \right]}} $$

The rate law for the ruthenium(III)-catalyzed reaction was derived similarly.
