# Supplementary material for: Mechanistic studies of uncatalyzed and ruthenium(III)-catalyzed oxidation of the antibiotic drug chloramphenicol by hexacyanoferrate(III) in aqueous alkaline medium: a comparative kinetic study
Source: Monatsh Chem. 2014 May 24;145(10):1561–73. doi: 10.1007/s00706-014-1208-7 (PMC4494844; doi:10.1007/s00706-014-1208-7)

**Mechanistic studies of uncatalyzed and ruthenium(III) catalyzed oxidation of the antibiotic drug chloramphenicol by hexacyanoferrate(III) in aqueous alkaline medium: a comparative kinetic study**

**M. D. Meti ● K. S. Byadagi ● S. T. Nandibewoor ● S. A. Chimatadar**

**SUPPLEMENTARY FIGURES**

**Suppl. Fig. 1** FT-IR spectra of p-nitrobenzaldehyde, the product obtained during the oxidation of chloramphenicol by hexacyanoferrate(III)


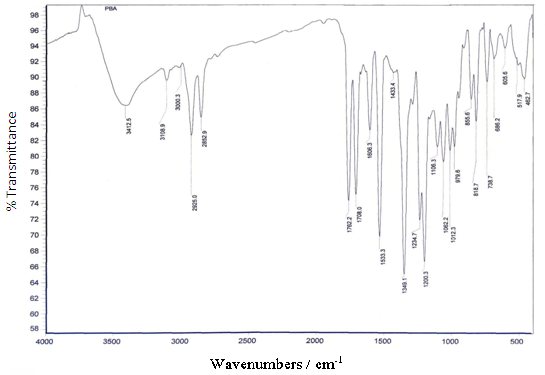


**Suppl. Fig. 2** Effect of ionic strength and dielectric constant for uncatalysed reaction

**Suppl. Fig. 3** Effect of ionic strength and dielectric constant for catalysed reaction

**Suppl. Fig. 4** UV-vis spectral changes during the oxidation of chloramphenicol by alkaline hexacyanoferrate(III) at 298K, [HCF] = 2.0 x 10^-4^, [CHP] =1.0 x 10^-3^, [OH^-^] = 0.5, and *I* = 1.10 mol dm^-3^ with scanning time interval of 1.0 min


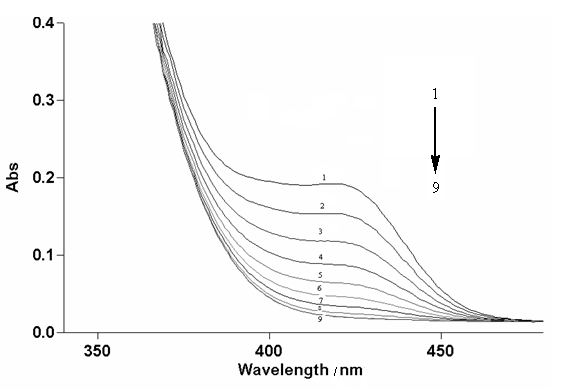

Supplement: Supplementary file 1 — Supplementary material 1 (DOCX 165 kb) [file 706_2014_1208_MOESM1_ESM.docx]
